# Encapsulation of Active Pharmaceutical Ingredients in Lipid Micro/Nanoparticles for Oral Administration by Spray-Cooling

**DOI:** 10.3390/pharmaceutics13081186

**Published:** 2021-07-31

**Authors:** Carmen S. Favaro-Trindade, Fernando E. de Matos Junior, Paula K. Okuro, João Dias-Ferreira, Amanda Cano, Patricia Severino, Aleksandra Zielińska, Eliana B. Souto

**Affiliations:** 1 Faculty of Animal Sciences and Food Engineering (FZEA), University of São Paulo, Rua Duque de Caxias, 225, Jardim Elite, Pirassununga 13625-900, Brazil; fematosjr@gmail.com (F.E.d.M.J.); paulaokuro@gmail.com (P.K.O.); 2 Department of Pharmaceutical Technology, Faculty of Pharmacy, University of Coimbra, Pólo das Ciências da Saúde, Azinhaga de Santa Comba, 3000-548 Coimbra, Portugal; j.dias.ferreira@outlook.pt; 3 Department of Pharmacy, Pharmaceutical Technology and Physical Chemistry, Faculty of Pharmacy and Food Sciences, University of Barcelona, 08028 Barcelona, Spain; acanofernandez@ub.edu; 4 Institute of Nanoscience and Nanotechnology (IN2UB), University of Barcelona, 08028 Barcelona, Spain; 5 Industrial Biotechnology Program, University of Tiradentes (UNIT), Av. Murilo Dantas 300, Aracaju 49032-490, Brazil; patricia_severino@itp.org.br; 6 Institute of Technology and Research (ITP), Av. Murilo Dantas, 300, Aracaju 49010-390, Brazil; 7 Institute of Human Genetics, Polish Academy of Sciences, Strzeszyńska 32, 60-479 Poznań, Poland; 8 CEB—Centre of Biological Engineering, University of Minho, Campus de Gualtar, 4710-057 Braga, Portugal

**Keywords:** spray chilling, congealing spray, spray cooling, lipid nanoparticles, oral drug delivery

## Abstract

Nanoencapsulation via spray cooling (also known as spray chilling and spray congealing) has been used with the aim to improve the functionality, solubility, and protection of drugs; as well as to reduce hygroscopicity; to modify taste and odor to enable oral administration; and many times to achieve a controlled release profile. It is a relatively simple technology, it does not require the use of low-cost solvents (mostly associated to toxicological risk), and it can be applied for lipid raw materials as excipients of oral pharmaceutical formulations. The objective of this work was to revise and discuss the advances of spray cooling technology, with a greater emphasis on the development of lipid micro/nanoparticles to the load of active pharmaceutical ingredients for oral administration.

## 1. Introduction

Encapsulation technology has stood out for decades to solve the limitations encountered in the delivery of active pharmaceutical ingredients (APIs), food ingredients, cosmetics, veterinary, hygiene, and cleaning products, among others. Among the most popular technologies used in the production of delivery systems for APIs, coacervation, solvent evaporation, solvent emulsion, ionic gelation, extrusion, high-pressure homogenization, spray drying, and spray cooling (also called spray congealing and spray chilling) have been particularly successful.

The spray cooling encapsulation technique consists of the preparation of a solution, dispersion, or emulsion of the APIs, followed by its atomization in a chamber in which cold air or liquid nitrogen is also injected [1,2]. Under these conditions, the API solidifies instantly, resulting in spherical particles, insoluble in water, and with a size range (micro- or nano-sized) governed by the configuration of the device used for atomization.

As encapsulating materials, natural, semi-synthetic, or synthetic mixtures are used, most often of lipid nature. Acids and fatty alcohols, triglycerides, and waxes with a high melting point are the most commonly used lipid materials for the loading of APIs in micro/nanoparticles by spray-cooling [3]. Such materials have been intensively used as pharmaceutical excipients, as they are low-cost products and are well tolerated physiologically; in addition, triacylglycerols are sensitive to lipases and have high biocompatibility, which minimizes the risks of acute and chronic toxicity [3,4,5].

The spray cooling technique is very convenient to encapsulate APIs, as it is a low-cost process; it is easy to scale up; and it is a non-stop, continuous approach and does not need organic solvents. The use of high temperatures may, on the other hand, be a limitation when thermosensitive APIs are to be processed, besides the risk of reduced encapsulation efficiency and expulsion of material from the chamber, thus reducing the yield of production.

There is no consensus in the literature regarding the nomenclature of the encapsulation technique by spray chilling, sometimes considered the synonym of spray cooling [6] and spray congealing [7,8,9,10]. With respect to spray chilling and cooling, the terms are usually used taking into account the melting point of the material selected as matrix. Thus, spray chilling is the process in which the encapsulating materials have a melting point between 32 and 42 °C, whereas in spray cooling, the encapsulation material usually has a higher melting point, between 45 and 122 °C [11,12,13,14]. The term spray congealing is used indiscriminately regardless of the melting temperature [9,15]. However, the lack of consensus on terminology does not overshadow the unique importance of this technology concerning encapsulation.

Several publications are available in the specialized literature describing the preparation of lipid micro/nanoparticles by the spray cooling technique for different purposes, which include changing the dissolution profile of poorly soluble APIs, prolonged release of APIs with a short half-life, alteration of taste and odor, increased stability of photosensitive ingredients, and encapsulation of bioactive proteins and peptides [16,17]. In a more recent approach, the spray cooling technique has been used for the production of lipid micro/nanoparticles for oral administration of APIs. However, not a single work has been found where these data have been compiled to provide an overview of the importance and the level of progress of this technique in the area, which is the primary aim of this review.

## 2. Encapsulation by Spray-Cooling

Spray-cooling consists of two stages. The first requires the addition of the API to be encapsulated to the matrix material, usually a melted lipid or an oil-in-water emulsion (in the case of hydrophilic APIs). The second step is the atomization of the lipid phase in the form of droplets, usually by a heated atomizer nozzle, to maintain adequate temperature; to avoid recrystallization of the lipid compounds; and, when this nebulized material comes into contact with a cooled environment (chamber with cold air injection or liquid nitrogen), with temperature below the melting point of the lipid, heat transfer occurs between the lipid and the cold air, leading to the solidification of the matrix and resulting in the formation of the particles [10,18].

**Figure 1:**
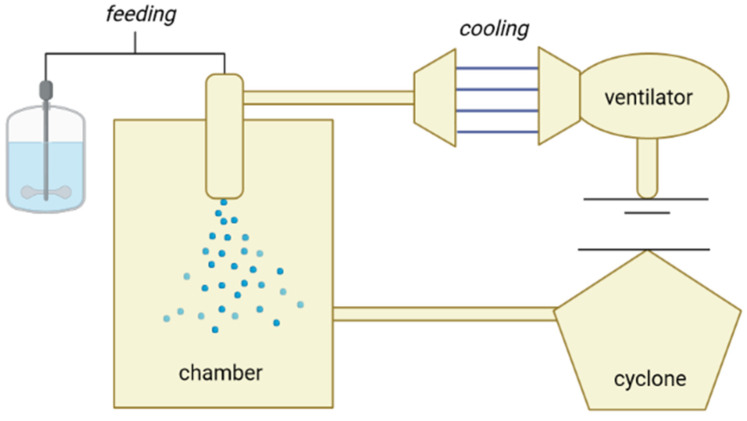
Schematic representation of a spray cooler.

The residence time of the sprayed droplets in the cooling chamber is short. The particles are collected in a container below the cooling chamber, while the very fine particles are transported by air to a cyclone, where they are collected in another container (Figure 1).

The production of particles by this method has been applied in several segments, such as pharmaceuticals, cosmetics, agricultural, veterinary, and food industries [19]. Like spray drying, the spray cooling encapsulation process has been reported for food ingredients, APIs, and flavourings [20].

Numerous applications for solid lipid particles have been investigated, for example, to modify odor and taste [21,22]; to protect the loaded material from conditions such as pH, enzyme action, moisture, oxygen, light, and optimization of the dissolution of poorly soluble APIs; to modify the release profile of loaded API; and to improve flow properties, handling, and appearance, among other purposes [10,23,24].

The process is widely used in the pharmaceutical field for the development of specialized API delivery systems [7,23,24]. In this context, spray congealing encapsulation may be related to the increased stability of sensitive compounds, such as small peptides like insulin, and poorly soluble drugs. The process also refers to the reduction of hygroscopicity, the alteration of undesirable flavours, and the alteration of the APIs’ release profile [25,26]. The generated product is insoluble and, generally, is released from the matrix in a controlled fashion until the melting point of the lipid matrix is reached [27]. The appropriate selection of the matrix material is crucial, as encapsulation can alter the properties of the API either by reducing its hygroscopicity and/or by increasing its stability [21].

Critical steps of the process include (i) atomizing the molten mixture (encapsulating agent and filling) and (ii) solidifying. The first refers to the disintegration of the melted mixture into small particles [28], while solidification is related to the process of recrystallization of the melted material into a solid by cooling it. From an operational point of view, insufficient cooling of the droplets leads to their agglomeration and/or adhesion on the surface of the chamber, an event that affects the morphology, the process itself, and other properties of the micro/nanoparticles.

Spray cooling is very similar to spray drying, mainly differing with respect to the direction of the energy flow involved. In the case of spray drying, energy is applied to the droplets, forcing the solvent to evaporate, while in the spray cooling, the energy is removed from the droplets, forcing the loaded API to solidify [18]. Thus, spray cooling can be regarded as a fusion between hot-melt technology (coating or agglomeration) and spray drying. Table 1 shows the differences between spray cooling and spray drying in several aspects. The initial configuration of spray cooling is very similar to spray drying; however, in contrast to spray cooling, the spray drying process enables the generation of small particles by rapid solvent evaporation from the surface of droplets. Conventional spray dryers can be used as spray coolers containing cold air. The instrument consists of two main parts, namely (i) a cooling chamber and (ii) the atomizer (Figure 1). For an efficient process, it is recommended that the dispersion of the melted mixture occurs within a very narrow melting temperature range, so that the particles are also solidified during spraying [29,30].

### 2.1. Process Variables

The variables with greater emphasis on this process include (i) the melting temperature of the lipid compounds; (ii) the temperature of the chamber; (iii) the temperature of the atomization air; (iv) the atomization air pressure; and (v) the feed flow of the melted mixture [10].

The critical conditions to obtain uniform and small particles include the low viscosity of the melted dispersion and the high atomization speed. It is suggested that the ideal viscosity is around 24 cP at 55 °C [29].

The performance of the spray cooler strictly depends on the atomization efficiency of the melted mixture. The efficiency of the atomization process, on the other hand, is directly associated with the different types of devices that can perform the atomization function. Such devices are atomizers, which can be of different types such as pressure nozzles, rotary or centrifugal, double fluid, and ultrasonic nozzles [10,18]. All available atomizers have recurring characteristics, such as the difficulty in nebulizing mixtures with higher viscosity. The conditions of supply and pressure used can be inferred under the homogeneous distribution of the particle.

Some studies have been carried out with the aim to investigate the influences of the process parameters. Maschke et al. found that increasing the atomization pressure from 5 to 6 bar decreases the size of the particles obtained by spray chilling [8]. Another important variable that influences the particle size is the viscosity of the feed mixture, which can be regulated through temperature or through the type and quantity of dispersed solids. Low viscosity (seen for higher temperature) results in smaller particle sizes [8], while the higher viscosity (owing to the addition of solids, for example) results in larger particle sizes [9]. The molten mixture, which will be the feed stream, has a characteristic solidification curve. When the atomized particles come into contact with the cooling medium, the material cools down to its solidification temperature. Thereafter, the temperature remains constant during the release of the product’s heat. Stable solid particles are then formed. Some products do not have a well-defined solidification point, with a phase change occurring over a range of temperatures, or the product may also turn into an amorphous solid without releasing the solidification heat, as a non-crystalline formation occurs. The particles can also be cooled below the solidification temperature, before hardening. The data on the physical properties of the melted material and its behaviour during the solidification process are important to define the design of the cooling chamber and the selection of the atomizer, as well as to determine whether the cooling should be carried out in one or two steps [31]. The atomized material goes through three cooling stages: (i) cooling the liquid; (ii) solidification; and, finally, (iii) cooling of the solid particles. The temperature of the nebulized droplets decreases as they come into contact with cold air. Upon reaching the solidification temperature, the droplets gradually solidify. As the newly solidified particles have a higher temperature than the environment, they continue to lose heat until they reach room temperature [10].

Another important aspect to ensure is the temperature control during the solidification process of the lipid wall, as its maintenance avoids the polymorphism in fats, a factor that directly affects the release profile of the loaded API. The lipid matrix can crystallize in different polyphonic forms. For example, in the case of rapid cooling, the lipid is preferably crystallized in an unstable form, α. On the other hand, when the cooling is slow, the tendency is the appearance of the form β [32,33].

Several studies have investigated the performance and improvement of equipment for the spray cooling technique, such as the use of pneumatic spray nozzles [9], compact ultrasound [34], and ultrasonic atomizer to disperse the atomized particles [35], among others. Ilić et al. studied the effect of some variables on the microencapsulation process by spray chilling [10]. The authors concluded that both the feed rate and the atomization pressure influence the final particle size. The higher the applied pressure, the smaller the particle size obtained and vice versa. With regard to feeding, a high feeding capacity increases the size of the particles, while, however, providing greater control of the particle size that can be achieved by controlling the atomization pressure.

### 2.2. Morphological Characteristics of Particles

Regarding the physical structure, the particles produced by spray cooling are of the matrix type, i.e., where the core or APIs are dissolved or dispersed in the crystallized lipid. In general, the particles produced have a spherical shape, which facilitates their flow [10], and are impermeable to water, but not resistant to it. These qualities are essential for a good incorporation of the material, owing to the reduction of the surface tension between the lipophilic surface of the particles and the aqueous environment where it will be added, allowing the flow of particles in the food [29].

As the process does not involve solvent evaporation, commonly observed in other techniques such as spray drying, the produced particles are usually dense and non-porous, in addition to being mechanically resistant, remaining intact under stirring [29].

For the pharmaceutical industry, the characteristics of the obtained particles, such as the smooth surface and mechanical resistance, are relevant as they improve the particles’ rheological behaviour, which is instrumental if they are aimed to fill, e.g., gelatine capsules or compression matrix in tablets’ production [29]. An additional coating can be applied to the microparticles obtained by spray cooling to ensure complete coverage of the particle and eliminate unwanted interactions surrounding them, during storage [30]. The particle size depends on several factors, such as (i) the filling material; (ii) the viscosity of the molten mixture; (iii) the disk configuration; and (iv) the rotational speed. Reactively to the operational configuration of the equipment, depending on the required particle size and the melting characteristics of the material, there is an adequate drop height [36].

### 2.3. Encapsulating Agents

Lipids are interesting alternatives as matrix material, owing to their ability related to the various morphological states such as, for example, emulsions, liposomes, and solid micro/nanoparticles [37,38]. The features of these materials that make them suitable for this purpose include (i) the ability to be stable under typical process conditions; (ii) the ease of atomization; and (iii) the moderate melting temperature, aiming at minimizing the degradation of the filling component [39]. Encapsulating materials can be divided, in general, between hydrophilic and lipophilic. In the pharmaceutical area, the former include polyoxylglycerides, poloxamers, polyethylene glycol (PEG), and polyethylene glycol esters, while lipophilics include beeswax; carnauba wax; cetearyl alcohol; cetyl palmitate; mono-, di-, and triacylglycerols (glyceryl behenate, glycerin palmitate stearate, glycerin stearate, glycerin palmitate); hydrogenated castor oil; microcrystalline wax; paraffin wax; stearic acid; and stearic alcohol [40]. The appropriate selection of the matrix material directly affects the modulation of the release of the encapsulated agent, and is decisive for the dissolving behavior of the APIs. Lipophilic matrices should be used to control the release of short half-life APIs, such as verapamil hydrochloride [41] and theophylline [42]. Hydrophilic materials should be used when the increase in the dissolution rate is achieved, such as carbamazepine [26], diclofenac sodium [43], and praziquantel [44]. The proper selection of the wall material is unique, as it affects several factors such as (i) the change in the properties of the encapsulated APIs; (ii) the increase in stability; (iii) control of the release profile; (iv) the change in unpleasant taste; and (v) the reduction of gastrointestinal irritation, enabling viability in certain environments [8,26,45,46].

### 2.4. Release Mechanisms

Controlled release aims at modulating the release of encapsulated APIs, being one of the most relevant properties made possible by microencapsulation. In response to a specific stimulus, that is, after the occurrence of a certain event, the particles start to release from its interior. The type and geometry of the core particles and, above all, of the matrix material, define the mechanism of release from the particle. Processes that employ hydrophilic matrix materials, in general, trigger a faster release of the nucleus, compared with those that use lipid compounds (fats or waxes), which tend to delay the release; while the hydrophilic materials release the API by diffusion, the release from lipophilic matrices is governed by erosion [47]. The release of APIs from the particles obtained by spray cooling occurs via erosion and leaching of the matrix. Some surfactants, depending on the type and concentration, can drastically affect the release rate of the API from the matrix. One study demonstrated that the addition of 4% of a non-ionic surfactant (sorbitan monooleate) resulted in an increase in the release rate from the lipid matrix; however, increasing the concentration of the surfactant to 10% led to a reduction in its release [30]. The release of APIs from lipid micro/nanoparticles can also be activated by the temperature. While in the release by matrix erosion, lipids are degraded by the action of lipases present in the body that participate in lipid metabolism, in the release triggered by temperature, the API is released responding to the change in temperature, affecting the physical state and the rate of release of the internal material. In this context, two distinct concepts stand out. The first is related to temperature sensitivity, relevant to materials that shrink or expand when a critical temperature is reached, and the second is due to melt activation, related to the melting of the wall material in response to the increase in temperature, such as is the case of walls made up of a modified lipid or waxes [48].

In the case of vitamins, potentially oxidizable compounds, micro/nanoencapsulation can, in addition to increasing stability, promote the alteration of possible flavours, or strange odours. In this case, APIs must be released after ingestion, i.e., in the stomach or intestine; for this purpose, lipid materials are generally used, although cellulose derivatives and cross-linked proteins can also promote the enteric release [49].

Regarding the concept of release after ingestion, for the effects to be truly understood, the microorganisms administered must remain alive when passing through the stomach, as the acidic environment and the presence of oxygen are harmful [50]. Therefore, the synchrony associated with the fact that lipid digestion (of the particle wall) occurs effectively in the intestine, where probiotics must act, reflects the efficiency and essential condition of using the technique.

The particles produced by this method may contain amounts of APIs that have not been effectively encapsulated, and can be attached to the outside of the lipid matrix. This situation leads to a high initial release of APIs, followed by a release that can occur through processes such as osmotic force, diffusion (even if small) of the filling through the matrix, mechanical breaks, and fusion of the lipids that make up the matrix [6]. In the case of lipid particles, there is an important caveat to be considered, as it is common to think that the release of APIs occurs only after the fusion of the matrix fat. However, this is not necessarily the only way that allows the release of APIs. As a significant number of APIs are located on the surface of the particles and have direct access to the environment, some burst release may occur. In addition, other factors influence the release kinetics, such as (i) osmotic force, (ii) the slow diffusion of water through the imperfections of the particles, and (iii) the mechanical rupture [6].

The use of surfactants directly affects the stability of solid lipid micro/nanoparticles. Their choice depends on the compounds involved in the production of the micro/nanoparticles as well as on the application of the final product [51,52].

The release profile of the APIs from the particles can also be influenced by changes in lipid matrices, concentration and type of surfactant (such as the presence of the lipophilic soy lecithin), and production parameters [53]. These surfactants are added to provide greater stability to the particles, as they assist in the effective connection between the filling and the matrix. Schubert et al. studied the incorporation of lecithin in the lipid matrix to optimize the incorporation of the filling [54]. These authors observed that the incorporation of APIs increased linearly with the increase in the concentration of lecithin present up to a certain percentage, and this effect was attributed to the formation of micelles in the middle of the lipid matrix, which promoted a higher loading of APIs. The addition of lecithin would prevent the formation of very crystalline structures, increasing the retention of the encapsulated material.

Zaky et al. studied the factors that affect the release kinetics of a protein encapsulated in microparticles composed of triglycerides prepared using the spray congealing technique [55]. The effect of particle size, particle morphology, and distribution on protein release was investigated by confocal laser scanning microscopy. It was firstly seen that water penetrates the microparticles and dissolves the incorporated protein, leading to the formation of water-filled pores, which allow its diffusion out of the matrix. It was also observed that the entry of water and the release of protein are strongly correlated processes, and attention was paid to the importance of three-dimensional analysis, enabling greater realism in the study of the distribution of the protein within the particle.

### 2.5. Advantages of the Process

Numerous advantages are associated with spray cooling, such as (i) speed, (ii) performance, and (iii) the relatively low cost of the process itself. As it does not require the use of water or organic solvents for its implementation, the elimination of residual solvents is not necessary. In addition to being considered a fast, safe, and reproducible physical process, it is also associated with an easy adjustment of particle size [42].

In the last two decades, spray cooling has also stood out on the environmental side, being considered an environmentally correct technique, and when compared with other procedures such as spray drying, it aims at lower energy and time consumption [56]. Another positive aspect is the ease of large-scale production, as it has the possibility of being operated continuously, eliminating production steps [57].

Particles with large sizes and various shapes, high APIs’ encapsulation efficiency, increased permeability characteristics through the oral mucosa, in addition to enabling satisfactory and desirable inject ability, are also reported in the process [56,58].

### 2.6. Disadvantages of the Process

Although lipid micro/nanoparticles have advantages, there are also some associated drawbacks, such as (i) the low encapsulation capacity of the material and (ii) the possibility of expulsion from the interior by the matrix. During the storage time of the particles, many lipid compounds applied for the formation of the dispersions can undergo changes, which can affect the API, its stability, and/or its release profile [59]. To overcome this limitation, the lipid material to be selected as a matrix compound should promote the solubility of the API, so that a higher loading can be achieved [60]. Besides, the selection of lipids that recrystallize in a metastable form, able to retain this polymorphic form longer, will also help to improve the loading parameters. Another limitation to be considered is the appropriate choice of the material to be encapsulated, as it must be stable at the melting temperature of its respective vector, which, in a way, restricts the API/matrix material relationship relevant to the condition of the technique. For many APIs, the observed degradation temperature is recurrently low, shortening the possibilities for the appropriate encapsulating agent. The issue of the use of melted mixtures must also be considered, which requires operational care, in order to avoid solidification and agglomeration of these, which compromises the atomization efficiency and, consequently, the process.

**Figure 2:**
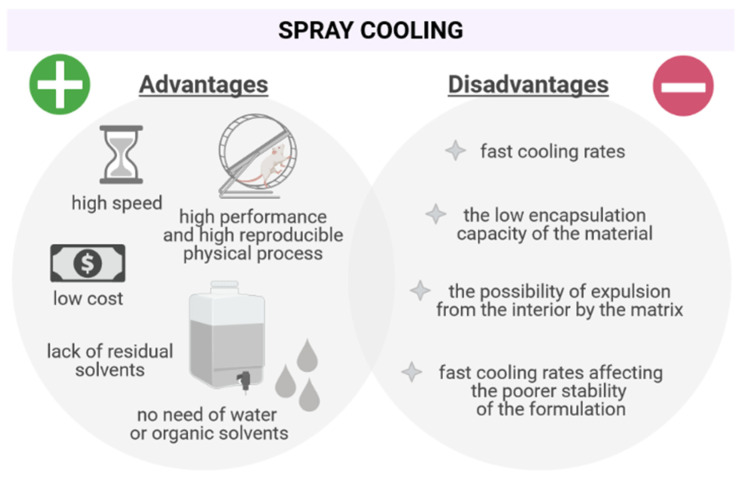
Summary of advantages and disadvantages of spray cooling.

The method has a drawback related to the fast cooling rates, which sometimes crystallizes the lipid matrix in a certain polymorphic form, such as the α arrangement, which is unstable, leading to the formation of disordered chains and/or with undesirable orientation and, consequently, promoting low barrier properties, and during shelf life is organised in more stable arrangements, resulting in the release of APIs [32,57]. Advantages and disadvantages of spray cooling are listed in Figure 2.

## 3. Encapsulation of APIs by Spray Cooling

Among the main techniques available for encapsulating APIs, the spray cooling technique has attracted special attention over the past 10 years. The use of lipid materials as matrix compounds, combined with the use of low temperatures and the absence of organic solvents during the process, are some of the advantages that have driven researchers to study the micro/nanoencapsulation of APIs by spray cooling as a way to overcome limitations in the pharmaceutical industry and to create new applications. The process is considered fast and of low cost, and has been tested for different purposes, which basically include improving the dissolution profile of poorly soluble APIs, the development of controlled release systems, the protection of APIs from adverse environmental conditions, alteration of flavour, and encapsulation of proteins and peptides with therapeutic potential by oral administration.

### 3.1. Improvement of the Dissolution Profile

Several publications have reported promising results in improving the dissolution rate of different APIs, including indomethacin [34], praziquantel [44], sodium diclofenac [43], glimepiride [10], piroxicam [61,62], and carbamazepine [26,63]. The improvement of the dissolution rate of these APIs has been obtained through the production of microparticles from solid dispersions prepared with matrices from hydrophilic polymers [26]. In the case of sodium diclofenac, for example [43], a 70% difference was found in the dissolution rate of the loaded APIs with gelucire 50/13 compared with its pure form. Positive results were also reported by Passerini et al. after assessing the dissolution rates of pure and microencapsulated carbamazepine with gelucire 50/13 [26]. According to the authors, the rate of dissolution of APIs in pure form was 40% after 10 min, and 80% in microencapsulated form. Later, these results were corroborated by other researchers who, using the same encapsulating agent, found that the solubility of microencapsulated carbamazepine increased 2.7 times in relation to pure APIs [63].

### 3.2. Sustained-Release Systems

By modifying the APIs’ release profile, several authors have developed pharmaceutical formulations for prolonged release of APIs that have a short half-life. In this case, unlike encapsulation aimed at enhancing the dissolution rate of APIs, the use of lipophilic matrices is recommended [34]. Theophylline [35,42], fembunfen [35], felodipine [64], and verapamil hydrochloride [41] are examples of APIs that achieved a modulated release by the spray cooling.

### 3.3. Increase of Stability

The spray cooling technique can also be applied to encapsulate APIs in order to increase stability in adverse conditions, such as the presence of light, oxygen, or unfavourable pH. Spray cooling can be used to protect probiotic microorganisms from the harsh gastrointestinal environment [65]. The use of these bacteria in both food and pharmaceutical formulations focuses on the need to maintain the viability of these microorganisms during the formulation of the product and over the storage period. The use of the spray cooling technique favours the microencapsulation of bacteria mainly because it does not subject the microorganisms to high temperatures or toxic solvents and lipid matrices are innovative vehicles to provide protection and the possibility of controlled release to probiotics.

### 3.4. Flavour Changes

Flavour change is often cited in the literature as one of the main purposes of micro/nanoencapsulation of APIs. However, few studies involving the spray cooling technique have been developed for this purpose to date. Some researchers have reported the microencapsulation of clarithromycin, a macrolide antibiotic with a strong bitter taste [66]. These authors verified the possibility of resorting to microencapsulation techniques with glyceryl monostearate and aminoalkyl methacrylate copolymer. In this way, it was possible to prevent the preparation from dissolving in the mouth, having an immediate release in the gastrointestinal tract, which resulted not only in flavour change, but also in better bioavailability.

### 3.5. Encapsulation of Proteins and Peptides

Another little-explored purpose, although very promising, is the microencapsulation of proteins and peptides through the spray cooling technique. Although the use of these components in pharmaceutical formulations has grown rapidly since 1980 owing to recombinant DNA technology, the development of delivery systems is still considered challenging because of the difficulties related to maintaining the stability and therapeutic action of proteins [67].

Several publications have addressed the use of microencapsulation for the development of controlled release systems of proteins and peptides of high therapeutic potential, including lactase [68], somatostatine [69], insulin [8], and growing hormones [70]. However, the choice of technique is limited by the need to maintain the integrity and bioactivity of the protein, and the use of high temperatures and the use of organic solvents are two factors that are directly linked to the protein denaturation process [71]. For this aspect, the use of the spray cooling technique can be advantageous, as there are no solvents or high temperatures. In addition, it is possible to use encapsulating agents of high biocompatibility, such as triglycerides, in formulations for parenteral release [8].

A study by Maschke et al. obtained positive results regarding the stability of insulin to the microencapsulation process by spray cooling [8]. The authors used glyceryl tripalmitate as a matrix material to obtain spherical particles with a smooth surface, with different concentrations of protein (0.5, 1%, and 2%), and sizes between 182.2 and 315 μm. Insulin was released for a period of at least 28 days, indicating a possible release up to six months, with the results being considered satisfactory and promising.

A successful attempt was made to encapsulate a high payload of bovine serum albumin (10 and 20% *w*/*w*) using low-melting lipids [72]. The microparticles produced were spherical in shape, sizes varied between 150 and 300 μm, and the encapsulation efficiency was greater than 90%. The studies to verify possible changes in the protein structure were carried out after encapsulation and the authors found that the structural integrity was maintained, supporting the conclusion that spray cooling is adequate to produce particles highly loaded with bovine serum albumin.

**Figure 3:**
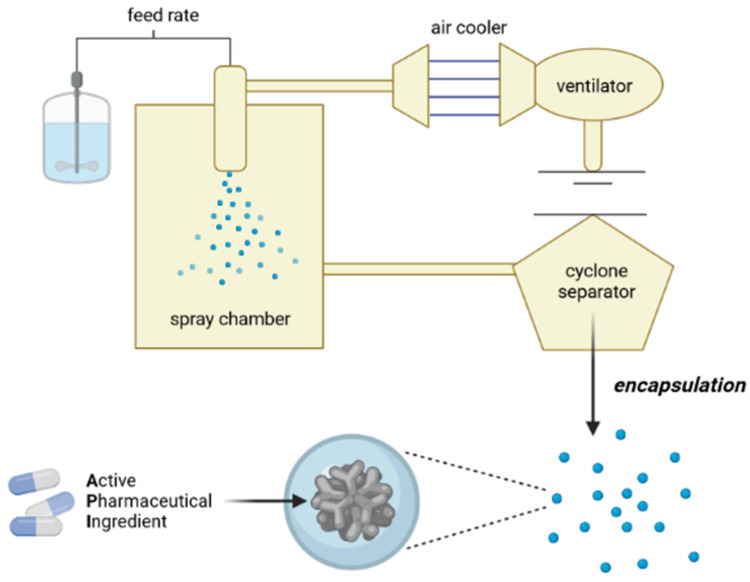
Encapsulation of APIs by spray-cooling.

The encapsulation procedure of APIs by spray cooling is illustrated in Figure 3.

## 4. Conclusions

Spray cooling encapsulation technology has been relatively little explored when compared with spray drying. However, it has the potential to be an excellent alternative for the encapsulation of several APIs, with the possibility of administration of particles through the oral route, providing controlled release, improved stability of the loaded API, and changing the unpleasant taste and flavour. The spray cooling technique has a high potential to overcome many other limitations presented in various industries. In the case of the pharmaceutical industry, it is possible that this technology is also useful for reducing the volatility and hygroscopicity of APIs, to facilitate the handling of toxic substances, the administration of incompatible APIs, and the reduction of gastric irritation. Although not a recent technique, the significant increase in work involving the micro/nanoencapsulation of APIs by spray cooling has only happened in the last 10 years. Many of the recently published works aimed to understand the process variables, as well as to propose changes in the equipment or procedures, in order to improve the performance in the preparation of the particles. It is very likely that, with the increased number of studies reporting the encapsulation of APIs by spray cooling, more researchers will become interested in the technique, thus enabling new applications in the pharmaceutical area.

## Figures and Tables

**Table 1:** Differences between spray cooling and spray drying techniques.

Parameters	Spray Drying	Spray Chilling
Energy flux	Energy applied to the droplets, forcing evaporation of the medium	Energy removed to the droplets, forcing the medium to solidify
Equipment	Feed tubes without heating	Heated feed tubes (to prevent solidification)
Flow in the equipment chamber	Hot air	Cold air or liquid nitrogen
Average particle size	5–150 µm	20–200 µm
Release mechanism	Dissolution	Difussion, heating
Morphology of particle	Particle with irregular geometry and porous surface due to solvent evaporation	Dense, spherical and smooth surface (absence of the evaporation effects of the solvent)
Coating	Water-soluble polymers	Waxes, fatty acids, water-soluble and water-insoluble polymers, monomers
Food ingredients	Vitamins, flavors, starter cultures, carotenoids, oils and fats, enzymes, acidulants	Ferrous sulphate, vitamins, minerals, acidulants
Steps in the process	(1) Disperse or dissolve the asset in the aqueous coating solution (2) Atomization (3) Dehydration	(1) Disperse or dissolve active in the melted lipid mixture (2) Atomization (3) Cooling

## Data Availability

Not applicable.
